# Facile fabrication of high-quality Ag/PS coaxial nanocables based on the mixed mode of soft/hard templates

**DOI:** 10.1038/srep30906

**Published:** 2016-08-01

**Authors:** Mimi Wan, Wenbo Zhao, Fang Peng, Qi Wang, Ping Xu, Chun Mao, Jian Shen

**Affiliations:** 1 National and Local Joint Engineering Research Center of Biomedical Functional Materials, School of Chemistry and Materials Science, Nanjing Normal University, Nanjing 210023, China

## Abstract

A new kind of high-quality Ag/PS coaxial nanocables can be facilely synthesized by using soft/hard templates method. In order to effectively introduce Ag sources into porous polystyrene (PS) nanotubes which were trapped in porous anodic aluminum oxide (AAO) hard template, Pluronic F127 (F127) was used as guiding agent, soft template and reductant. Meanwhile, ethylene glycol solution was also used as solvent and co-reducing agent to assist in the formation of silver nanowires. The influences of concentration of F127 and reducing reaction time on the formation of Ag/PS coaxial nanocables were discussed. Results indicated that the high-quality Ag/PS coaxial nanocables can be obtained by the mixed mode of soft/hard templates under optimized conditions. This strategy is expected to be extended to design more metal/polymer coaxial nanocables for the benefit of creation of complex and functional nanoarchitectures and components.

One-dimensional (1D) nanoarchitectures including nanorods, nanowires, nanoribbons, and nanotubes are expected to play a critical role as both interconnected and functional units in the fabrication of nanodevices such as nano-motor and nano-optoelectronic devices^1^,^2^. To integrate these above nanostructures into some higher performance devices, there are great demands to design and create complex and functional nanoarchitectures and components. Recently, many efforts have been devoted to the fabrication of coaxial nanocables with controlled size and shape because of their unique structure and novel properties compared with those of their single-component nanowires^3^,^4^,^5^,^6^,^7^,^8^,^9^,^10^. Some chemical and physical synthetic methods for coaxial nanocables including the template covering method, template filling method, and simultaneous synthesis method have been investigated^11^,^12^. For instance, some researchers reported the preparation of polystyrene (PS) and silica-coated Au nanorods. PS coating of Au nanorods was accomplished by the emulsion polymerization of styrene in the presence of cetyltrimethylammonium bromide (CTAB)-coated Au nanorods^13^. However, there were two aspects needed to be improved for their reaction system. One was that, it was not all the gold nanorods can be wrapped by PS sheath. Another was that, some adhesive gold nanorods were encapsulated together by PS sheath. The same situation also occurred in the hydrothermal system for synthesizing of Ag/C nanocables designed by other groups^3^. Many other reported works about the preparation of coaxial nanocables often relied on harsh conditions such as high temperature (e.g. annealing process under high temperature of 600–1000 °C), several complex procedures, and so on^14^, which greatly limited their wide application.

Template-assisted method is the most commonly used method among all fabrication methods for 1D hybrid nanostructures due to its conceptually simple, intuitive, straightforward, and versatile route^15^. There are two types of templates including soft (organic compounds) and hard (channels in solids) ones. For the former, more complex architectures of 1D hybrid materials will be created because of the complex self-assembly process of organic compounds, especially when more different blocks or non-linear topologies are involved^16^. As to the latter, the specific independent channels in solids can greatly improve the adhesion efficiency of current preparation methods for nanocables. Nonetheless, how to effectively guide the metal solution into the channels in solids became a great challenge in using this method to prepare the metal/polymer coaxial nanocables^17^. Meantime, it is also difficult to obtain the continuous linear metal axis after specific reduction reactions of metal salt.

Herein, we report a one-step synthetic strategy based on the mixed mode of soft/hard templates for efficient synthesis of high-quality Ag/PS coaxial nanocables. Ag nanowires acted as cable axis, which was obtained by spiralization and reduction of AgNO_3_ that driven by soft template from self-assembly of Pluronic F127 and ethylene glycol. While the insulated outer sheath PS was formed by anodic aluminum oxide (AAO) hard template. During the preparation process, F127 acted as guiding agent, soft template and reductant for AgNO_3_ solution. Meanwhile, ethylene glycol solution was used as solvent and co-reducing agent to assist in the formation of silver nanowires. The optimized conditions for preparing of high-quality Ag/PS coaxial nanocables were investigated. The high quality of Ag/PS coaxial nanocables we prepared was reflected in their neat Ag nanowires wrapped with the PS nano-sheath with independent morphology and uniform thickness. Moreover, the high ratio of conductive Ag core radius/insulating PS sheath thickness was an interesting feature compared with coaxial nanocables obtained by other literatures.

## Results

### Synthesis of PS nanotubes

**Figure 1:**
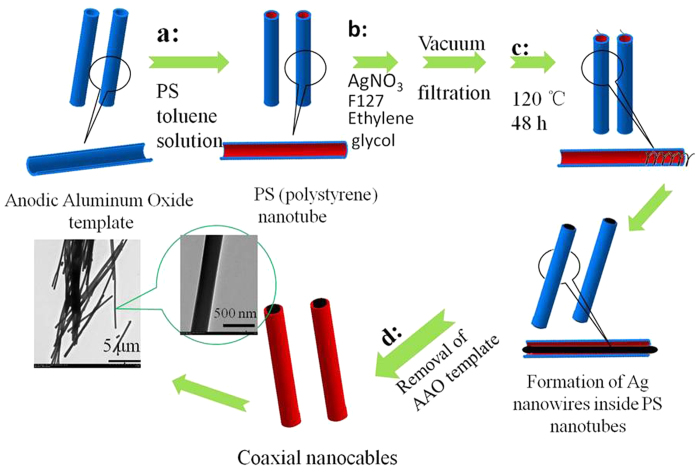
Schemetic illustration of the formation of Ag/PS coaxial nanocables based on the mixed mode of soft/hard templates.

Porous AAO template synthesis is one of the most commonly used methods because that the walls of AAO template exhibit high surface energy. Polymer nanotubes can be obtained by wetting the porous AAO templates with polymer or solutions. In this work, the porous AAO membrane (Whatman Int. Ltd, England) with pore size of about 200 nm was used as hard template. PS-toluene solution entered the pores of the AAO template, and the thin surface film was formed to cover the pore walls of AAO owing to the fact that cohesive driving forces for complete filling are much weaker than the adhesive forces^18^. After being treated with the solvent evaporation and template surface polishing processes, PS nanotubes in AAO template were obtained (Figs 1a and S1). This method used to fabricate the PS nanotubes is simple and reproducible. As shown in Fig. S1a,b, SEM images of AAO template exhibited pores of around 200 nm. SEM and TEM images of PS nanotubes without AAO template are shown in Fig. S1c,d, which displayed the thickness of PS nanotubes was about 30–40 nm.

### Synthesis of Ag/PS coaxial nanocables

The AgNO_3_-ethylene glycol solution was difficult to be imported into PS nanotubes in AAO template due to the hydrophobicity and nanosized effect of PS nanotubes. So how to effectively guide the metal solution into the PS nanosized channels became the first key to prepare high-quality Ag/PS coaxial nanocables. Two methods were used to improve the guiding efficiency of the solution entering into the PS nanotubes (Fig. 1b). One was that the solution containing silver sources was introduced into AAO template with PS nanotubes by vacuum filtration method. Another was that guiding agent was adopted to introduce silver solution into PS nanotubes. F127, PEO(98)-PPO(67)-PEO(98) triblock copolymers (MW approximately 12600, BASF Co. Ltd., Germany), which had hydrophilic PEO blocks and hydrophobic PPO blocks, was chosen as guiding agent. F127 made it easier for AgNO_3_-ethylene glycol solution to enter the PS nanotubes, therefore, it is called guiding agent in this case. In addition, the amphiphilic PEO blocks also played roles of soft template agent and reductant for AgNO_3_ solution^4^.

After AgNO_3_ solution was introduced into PS nanotubes, how to obtain the continuous linear Ag axis in PS nanotubes during reduction process became the second key to synthesize high-quality Ag/PS coaxial nanocables. The phenomena of non-continuous metal nano-dots or nano-segments often appeared in the previous researches about inorganic/organic hybrid nanotubes^18^. In this case, the mixture was treated under 120 °C for 48 h, the reduction reaction of silver nitrate occurred in the presence of guiding and reducing agent (Fig. 1c). After a period of reaction, Ag nanowires inside the PS nanotubes were formed. Finally, the AAO template was removed by NaOH solution, and the Ag/PS coaxial nanocables were obtained (Fig. 1d).

### Optimized synthesis conditions of Ag/PS coaxial nanocables

**Figure 2:**
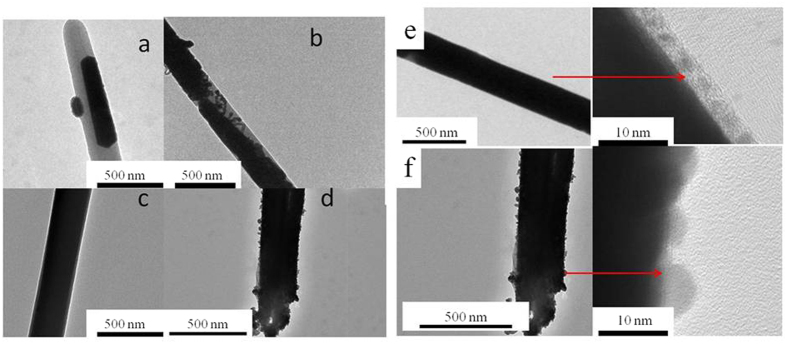
(left) TEM images of samples (**a**) PAF/0/48, (**b**) PAF/0.05/48, (**c**) PAF/0.1/48, and (**d**) PAF/0.2/48. (right) Higher magnification of TEM images of samples (**e**) PAF/0.1/48, and (**f**) PAF/0.2/48.

The optimization of preparation conditions for high-quality Ag/PS coaxial nanocables is necessary. Firstly, the reducing time for the AgNO_3_-ethylene glycol solution imported into the PS nanotubes was set to 48 hours, and then the influence of F127 content on the quality of nanocables products was investigated. As shown in Fig. 2, when the content of F127 was 0 g ml^−1^ (PAF/0/48) and 0.05 g ml^−1^ (PAF/0.05/48), Ag materials cannot fully filled PS nanotubes (Fig. 2a,b). When the concentration of F127 increased to 0.1 g ml^−1^, ideal filled Ag nanowires inside the PS nanotubes were observed (Fig. 2c). However, PS nanotubes were destroyed quite a lot with further increase of F127 concentration (Fig. 2d). Under higher magnification, a neat Ag nanowire wrapped with the PS nanolayer with uniform thickness (about 5 nm) was observed from the PAF/0.1/48 sample (Fig. 2e). As for the PAF/0.2/48 sample, the irregular breakage of the PS sheath was found (Fig. 2f). As shown in Fig. S1, the thickness of PS nanotube was about 30–40 nm, which was thicker than that of the PAF/0.1/48 sample. The decrease of the PS thickness can be attributed to the following facts: (1) The reducing temperature (120 °C) of silver nitrate was higher than the glass transition temperature (about 100 °C)^19^, (2) PS nanotubes are in glassy state under the reducing temperature and will become thinner owing to the squeezing of the reduced Ag sources, resulting in the decreased thickness of the PS layer of the final nanocables, which was in consistent with the results reported by other literatures stated that the PS film thickness would decrease under glassy state^20^.

These results indicated that: (1) Silver sources were difficult to enter into PS nanotubes without addition of F127, and partially reduced Ag product that not fully filled PS nanotubes was found. (2) The concentration of F127 played a decisive role as guiding agent for the introduction of AgNO_3_-ethylene glycol solution entering into the PS nanotubes, which may be attributed to its amphiphilic property. (3) Excessive F127 (0.2 g ml^−1^) led to the breakage of PS nanotubes, which cannot get high-quality Ag/PS coaxial nanocables. The F127 concentration of 0.1 g ml^−1^ is suitable for this preparation system.

Meantime, results obtained from UV-Vis spectra confirmed the results of TEM images of the PAF/m/48 (m representing the concentration of F127). Figure S2 depicted the UV-Vis spectra of PAF/m/48 samples with different concentration of F127, which showed a broad band in the wavelength range of 350–400 nm. Long-wavelength band can be attributed to the presence of Ag nanowires^4^,^21^, whose intensity increased with the concentration of F127. There is no obvious adsorption band in the spectrum of PAF/0/48, indicating that the nanowire morphology of Ag in this sample was incomplete. And the adsorption band weakened a lot when the concentration of F127 reached at 0.2 g ml^−1^, implying that excess amount of F127 may not promote the formation of high-quality Ag nanowires.

**Figure 3:**
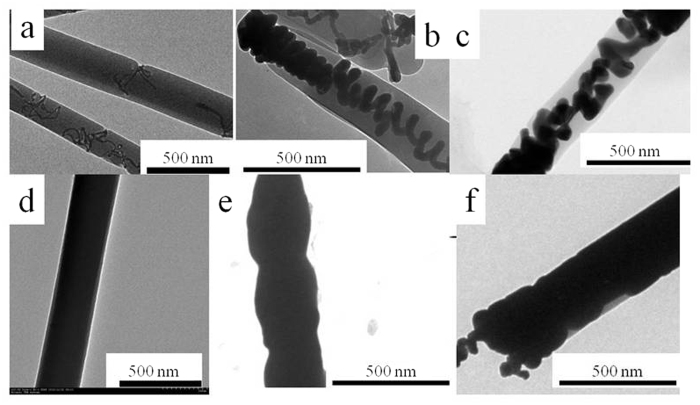
TEM images of samples (**a**) PAF/0.1/12, (**b**) PAF/0.1/24, (**c**) PAF/0.1/36, (**d**) PAF/0.1/48, (**e**) PAF/0.1/60 and (**f**) PAF/0.1/72.

The effect of reduction time on the quality of Ag/PS coaxial nanocables was also investigated based on the F127 concentration of 0.1 g ml^−1^. PAF/0.1/n samples with different reducing time were prepared. Figure 3 (TEM images) illustrated the control of reducing time on the morphology of Ag nanowires. As has been widely regarded, F127 played an important role in controlling the growth of Ag nanowires as growth directing agent and assistant reductant^22^. When the reduction time is 12 hours, a few discontinuous Ag nanowires entrapped into the PS nanotubes were found (Fig. 3a), and when the reaction time increased to 24 and 36 hours, the spiral continuous Ag nanocoil inside PS nanotubes were observed (Fig. 3b,c). Until 48 hours, the Ag nanorods filled PS nanotubes, and ideal Ag/PS coaxial nanocables were obtained (Fig. 3d). Figure S3 showed the UV-Vis spectra of these samples. It can be observed that band located at 350–400 nm was not obvious until the reducing time reached to 48 h, and no adsorption band can be seen when the reducing time is 12 h. What’s more, there still existed an obvious adsorption band in the UV-Vis spectrum when the PS layer was dissolved by ethyl acetate for PAF/0.1/48 sample (Fig. S3), verifying the existence of Ag nanowires. TEM images of PAF/0.1/60 and PAF/0.1/72 were also shown in Fig. 3. It can be observed that excessive Ag sources will be formed for the reducing time of 60 h and 72 h. As a result, the PS layer will be destroyed by the excessive Ag sources to some extent, which cannot get high-quality Ag/PS coaxial nanocables. Therefore, the reducing time of 48 h is suitable for this preparation system.

In order to further verify the nanowire morphology of Ag inside the PS nanotube, PS sheath was dissolved by ethyl acetate. TEM images of the samples without PS nanotubes were shown in Fig. S4. It can be observed in Fig. S4 that PAF/0.1/12 exhibited scattered Ag nanowires morphology, and PAF/0.1/24 sample showed incomplete Ag nanowire morphology. Meantime, PAF/0.1/48 without PS nanotube can still maintain its ideal nanowire morphology. Moreover, high resolution transmission electron microscopy (HRTEM) images illustrated that the spacing between two adjacent lattice planes was about 0.25 nm, 0.24 nm, and 0.22 nm, which is in consistent with the spacing of the Ag crystal^23^,^24^.

**Figure 4:**
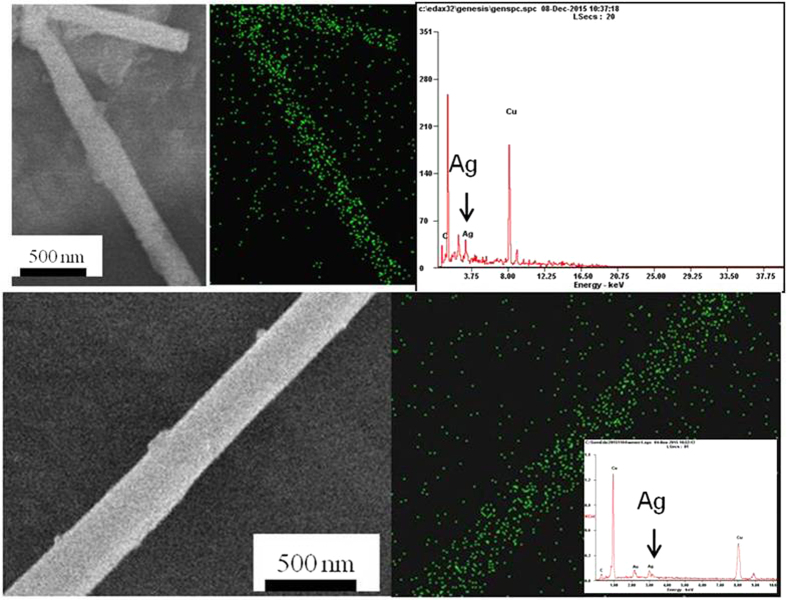
SEM and EDX mapping image of PAF/0.1/48 sample.

To prove that the Ag had actually formed as a continuous phase, SEM element mapping of PAF/0.1/48 sample was performed. As is shown in Fig. 4, bright field electron-diffraction contrast images and elemental map of Ag can be observed in the SEM mapping images of the sample. The distribution of Ag element in the sample was very dense and regularly arranged, verifying the continuous phase of the silver element. Also, EDS spectra of the sample confirmed the existence of Ag element (Fig. 4).

## Discussion

**Figure 5:**
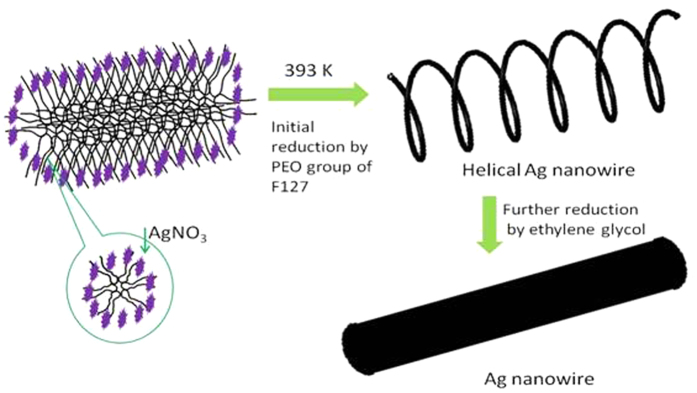
Schemetic illustration of the growth process of Ag nanowires that entrapped into PS nanotubes.

In previous studies, many kinds of materials have been synthesized through the helical assembly of polymers and nanowires, which are inspired by helical structure in plants^25^,^26^,^27^. Here, F127 plays a role of soft template which can help the small Ag segments self-assemble into the spiral Ag nanocoil entrapped into the PS nanotubes (Fig. 5). As AgNO_3_-ethylene glycol solution was difficult to be imported into the PS nanotubes, F127 was used to facilitate AgNO_3_- ethylene glycol solution to enter into the PS nanotubes. In order to verify our assumption, contact angles of the precursor solution of PAF/n/48 with or without F127 and F127 water solution on PS thin film were examined (Fig. S5). It can be seen from Fig. S5 that when the concentration of F127 solution increased from 0 to 0.1 g ml^−1^, contact angle decreased from 86 to 43 degree. And for the solution with ethylene glycol as solvent, the contact angle decreased from 60 to 31 degree, illustrating that F127 can greatly facilitate AgNO_3_ - ethylene glycol solution to enter into the PS nanotubes. Moreover, owing to its particular amphipathic nature^28^,^29^, F127 would undergo self-assembly process to form micelles with PEO as shell and PPO as core in AgNO_3_ - ethylene glycol solution^30^. Generally, PEO group can reduce AgNO_3_ at about 100 °C^4^, while the reduction temperature of AgNO_3_ by glycol was usually higher than 120 °C^31^. As a result, Ag sources would be reduced by PEO group below the temperature of 120 °C, and the products would locate in the layer of PEO domains. Hence, helical Ag nanowires were firstly formed at the beginning of the reducing process (Fig. 3a,b)^32^. With the increase of the reducing time, further reduced Ag caused by ethylene glycol would be formed to fill the helical space of the Ag nanowires. Therefore, Ag nanowires were formed in the final product (Figs 3d and 5).

In order to examine whether F127 played important role in the formation of helical morphology and the reducing ability of ethylene glycol at 120 °C, additional experiment has been carried out. Ag/PS nanocables (denoted as PAF/0/n, where n represents different reducing time) without addition of F127 are synthesized. TEM images of PAF/0/4 and PAF/0/24, of which the reducing time were 4 h and 24 h, were shown in Fig. S6. It is observed in Fig. S6 that no helical morphology can be found without F127, and discontinuous Ag nanoparticles were found on PAF/0/4 sample. When the reducing time increased to 24 h, Ag nanowires can be formed in some PS nanotubes. However, not all the PS nanotubes can be filled with Ag nanowires, illustrating that Ag/PS nanocables cannot be realized by single reduction of ethylene glycol.

As demonstrated above, we used a simple strategy to combine PS nanotube synthesis with Ag nanowire preparation in this paper, obtaining novel kind of metal/polymer coaxial nanocables. The synthesis method used in this work can produce much cleaner and independent nanocables without extra separation and purification processes, avoiding the phenomenon of adhesive nanocables appeared in many other literatures^3^. The key to this success was the new concept of a hard conductive core wrapped with soft insulating shell. Unlike many nanocables reported by literatures, in which the protective shell are mostly carbon material prepared by carbonization process^5^,^11^, this new kind of nanocable with polymer as protecting shell can offer much more choices for design of novel nanocables owing to large amount of the selective polymers. What’s more, it is quite possible to modify the surface properties of the nanocables by selecting different kinds of polymer shell.

Furthermore, the ratio of the conductive layer to the insulative layer was very important to the electric property of the nanocables. The ratios of Ag radius/C thickness of the nanocables reported by many literatures were often about 1:1 (100 nm:100 nm) or even lower^3^,^14^, which was not beneficial for the conductive property of the nanocables. When the insulating layer was replaced by PS nanotube, the ratio of Ag nanowire radius to PS sheath thickness was increased to about 20:1 (100 nm : 5 nm).

Meantime, this new method to synthesize coaxial nanocables can greatly lower the synthesis temperature in comparison with traditional preparation methods. The whole procedure was performed at 120 °C or below, which was much lower than the commonly used temperature in other lituratures (higher than 160 °C or even higher than 500 °C)^9^,^33^.

## Conclusion

In summary, we have developed a facile and versatile strategy to construct well-defined nanocables with Ag nanowires as comducting cores and PS as the insulator through helical assembly of Ag nanowires driven by self-assembly of F127. The synthesized strategy used in this paper contains several advantages: i) For the first time, F127 is used not only as guiding agent, but also as soft template and reductant, to benefit the introduction of Ag sources into PS nanotubes and the reduction processes. ii) Compared with coaxial nanocables obtained by other literatures, much cleaner and independent nanocables are produced in this paper without extra separation and purification process. iii) The synthesis temperature can be greatly lowered to 120 °C or below. iv) High quality of the coaxial nanocables with high ratio of conductive core has been obtained, and the ratio of Ag nanowire radius to PS sheath thickness is increased to 20:1 from common value of 1:1. Unlike many nanocables reported by literatures, in which the protective shell are mostly carbon material, this new kind of nanocable with polymer as protecting shell can offer much more choices for design of novel nanocables owing to large amount of the selective polymers. The Ag/PS coaxial nanocables have potential applications in nanodevices for the purposes of conserving energy and miniaturization.

## Methods

### Preparation of Ag/ PS coaxial nanocables

15 μL of the PS solution (5% in toluene) was dropped on Anodic Aluminum Oxide (AAO) template with diameter of 200 nm (Shanghai Ligen Trading Co. Ltd.). After the solvent completely evaporated for 1 h, the AAO/PS composite was polished for about 15 min.

The introduction of Ag sources into AAO/PS composite was achieved by vacuum filtration of AgNO_3_ precursor. A solution containing AgNO_3_ (0.5 g mL^−1^), ethylene glycol (5 mL), and different amount of F127 water solutions (0, 0.05 g ml^−1^, 0.1 g ml^−1^, 0.2 g ml^−1^) were prepared for further use. Then the solution was placed on the top side of the AAO/PS composite and a vacuum was applied to the bottom of the membrane for about 20 min until the entire volume of the solution was pulled through the membrane. The obtained composite was treated under 120 °C for given time (12 h, 24 h, 36 h, 48 h). Finally, the composite was put into 3 M sodium hydroxide to remove the AAO template. The obtained samples were named as PAF/m/n, where m represented the concentration of F127 and n represented the reducing time.

### Characterizations

Scanning electron microscopy (SEM) images of the samples were obtained by using a Hitachi S4800 FE-SEM system with 10 kV accelerating voltage and 10 mA of beam current. Transmission electron microscopy (TEM) analysis was carried out on a JEM-2100 electron microscope operating at 200 kV. UV-vis spectra of samples were collected on a UV scanning spectrophotometer (Cary 50) equipped with an integrating sphere from 200 to 800 nm. Static contact angles were measured using the sessile drop method with a 3 μL certain liquid droplet and a telescopic goniometer (Rame-Hart, Inc., Mountain Lake, NJ).

## Additional Information

**How to cite this article**: Wan, M. *et al*. Facile fabrication of high-quality Ag/PS coaxial nanocables based on the mixed mode of soft/hard templates. *Sci. Rep.* **6**, 30906; doi: 10.1038/srep30906 (2016).

## Supplementary Material

Supplementary Information
