# Supplementary material for: Facile fabrication of high-quality Ag/PS coaxial nanocables based on the mixed mode of soft/hard templates
Source: Sci Rep. 2016 Aug 1;6:30906. doi: 10.1038/srep30906 (PMC4967888; doi:10.1038/srep30906)
Supplement: Supplementary Information [file srep30906-s1.pdf]

Supporting Information

Facile fabrication of high-quality Ag/PS coaxial nanocables based on the mixed mode of soft/hard templates

Mimi Wan, Wenbo Zhao, Fang Peng, Qi Wang, Ping Xu, Chun Mao*, Jian Shen*

National and Local Joint Engineering Research Center of Biomedical Functional Materials, School of Chemistry and Materials Science, Nanjing Normal University, Nanjing 210023, China, +86 25 85891651

E-mail: (maochun@njnu.edu.cn (C. Mao), jshen@njnu.edu.cn (J. Shen))

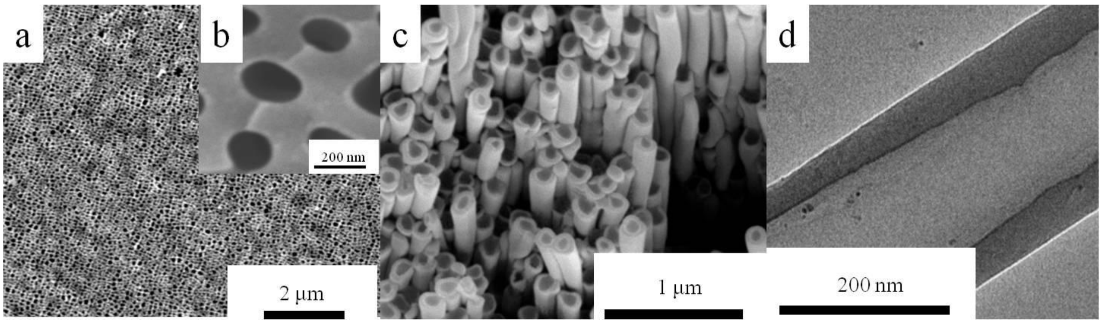

Supplementary Figure S1 SEM images of (a) and (b)AAO template, (c) PS nanotubes without AAO template and (d) TEM image of PS nanotube.

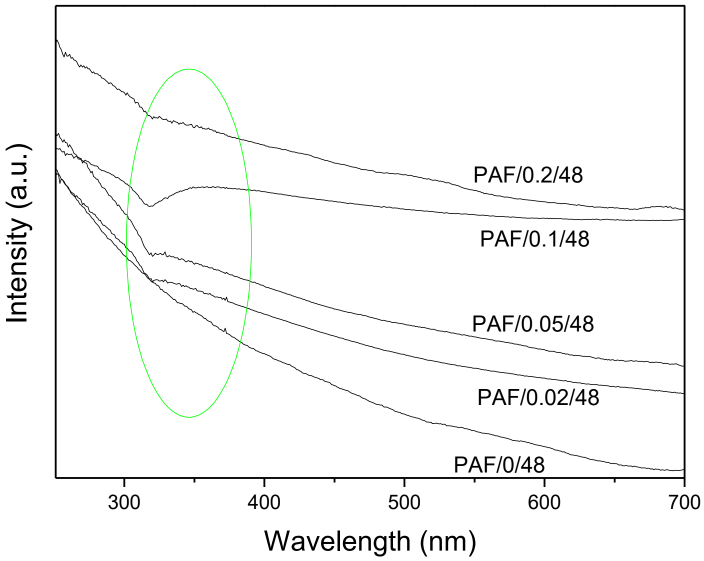

Supplementary Figure S2 UV-Vis spectra of the PAF/m/48 samples with different concentration of F127.

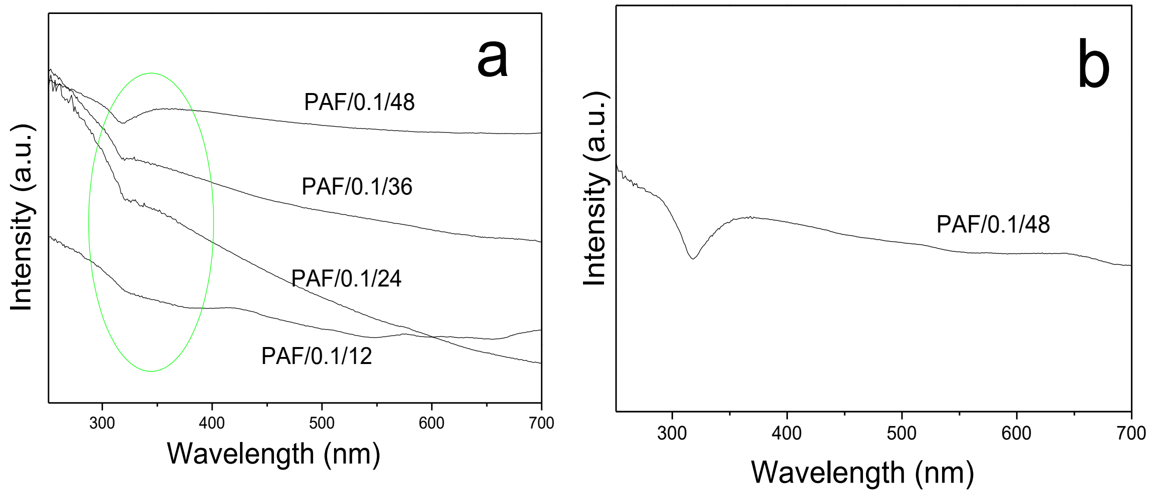

Supplementary Figure S3 UV-Vis spectra of (a) the PAF/0.1/n samples with different reducing times, and (b) PAF/0.1/48 without PS.

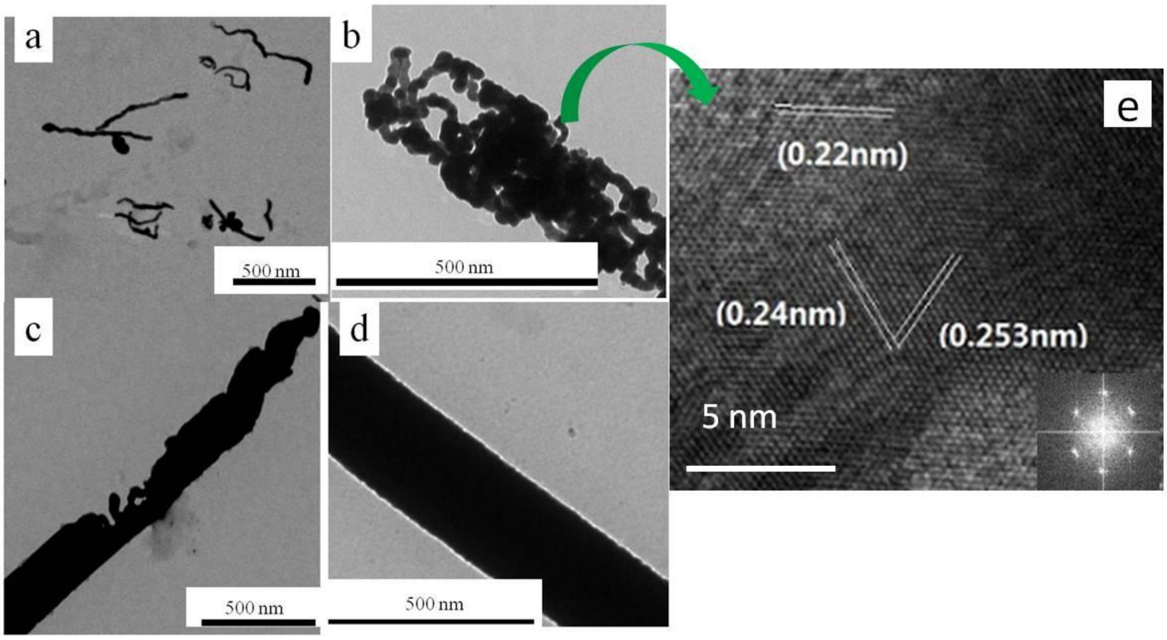

Supplementary Figure S4 TEM images of samples (a) PAF/0.1/12, (b) PAF/0.1/24, (c) PAF/0.1/36, (d) PAF/0.1/48 after dissolving of PS layer, and HR-TEM image shows the lattice fringes of silver nanowire (e).

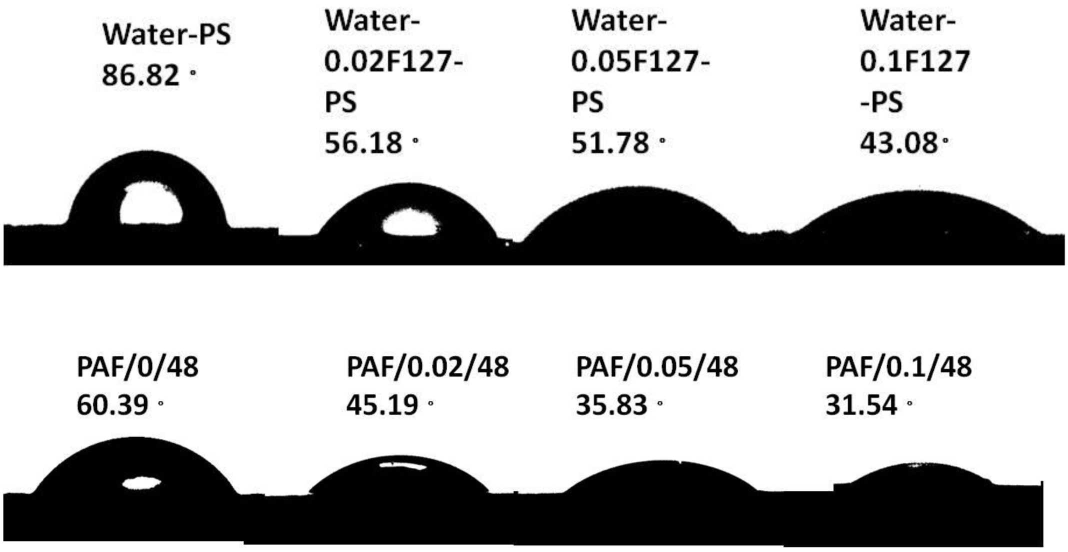

Supplementary Figure S5 Contact angle of the PAF/n/48 with PS film.

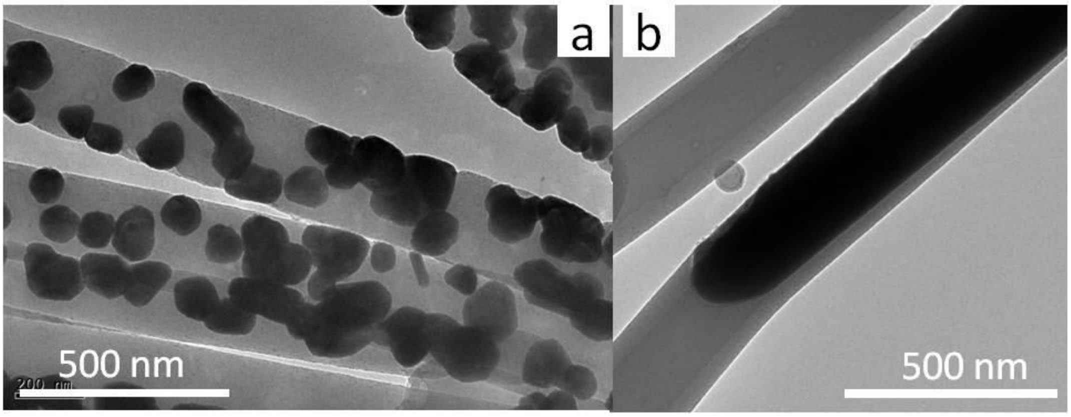

Supplementary Figure S6 TEM images of PAF/0/n samples without addition of F127: (a) PAF/0/4 and (b) PAF/0/24.
